# The Hidden Burden of Parkinson's Disease in Cambodia: A Call to Action

**DOI:** 10.1002/puh2.70343

**Published:** 2026-08-07

**Authors:** Virak Sorn

**Affiliations:** ^1^ Faculty of Health Sciences and Biotechnology University of Puthisastra Phnom Penh Cambodia

**Keywords:** Cambodia, noncommunicable diseases, Parkinson's disease, quality of life, universal health coverage

## Abstract

Parkinson's disease (PD) is an emerging global public health concern, particularly in low‐ and middle‐income countries (LMICs), yet it remains under‐recognized in Cambodia. The absence of reliable epidemiological data, limited specialist workforce, delayed diagnosis, and inadequate access to essential medicines contribute to a substantial but largely hidden disease burden. Health system limitations, such as inadequate primary care capacity, rehabilitation services, and insufficient community‐based support, further exacerbated disparities in PD care. This article highlights critical gaps in PD management in Cambodia and calls for urgent, coordinated action. Key priorities include establishing national surveillance systems, strengthening primary healthcare, expanding the neurology workforce, building local capacity, promoting cross‐cultural education, leveraging digital health and telemedicine, improving access to essential medicines, and integrating PD into national health policies as core components of Cambodia's long‐term strategy for enhancing PD care. Addressing these challenges is essential to ensure equitable and effective care for individuals living with PD and to align Cambodia's health system with universal health coverage (UHC) goals.

## 1 Introduction

Parkinson's disease (PD) is the second most common neurodegenerative disorder globally, with a rapidly increasing burden, particularly in low‐ and middle‐income countries (LMICs), which account for approximately 75% of total cases [1, 2]. Global projections estimate that PD prevalence will reach 267 cases per 100,000 population by 2050, representing a 76% increase from 2021, whereas age‐standardized prevalence is expected to rise by 55% to 216 per 100,000 [3]. Although high‐income countries have made substantial progress in diagnosis and management, LMICs continue to face persistent challenges in responding to the growing burden of PD [3, 4].

**FIGURE 1:**
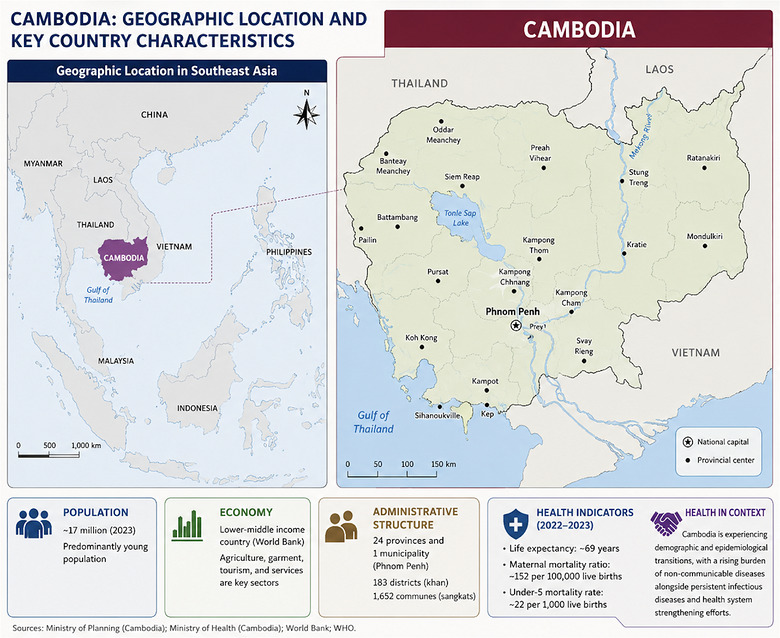
Cambodia's geography and a summary of the healthcare system characteristics.

Cambodia, an LMIC geographically located in mainland Southeast Asia (Figure 1), is characterized by a predominantly Khmer‐speaking population, cultural diversity, and a mixed healthcare system comprising public, private, and nongovernmental sectors. As the country undergoes rapid demographic and epidemiological transitions, the burden of noncommunicable diseases (NCDs), including neurological disorders, continues to increase [5]. Despite this growing burden, neurological conditions, such as PD, remain largely overlooked within the national health agenda. Cambodia lacks population‐based epidemiological data and a national PD registry, resulting in substantial underdiagnosis and underreporting of the disease. Consequently, many patients present at advanced stages of illness, whereas access to timely diagnosis and comprehensive care remains fragmented, particularly in rural and underserved areas where healthcare resources and specialist services are limited [6, 7, 8]. These challenges are compounded by resource constraints, workforce shortages, and inequities in service delivery, contributing to a substantial “hidden burden” of disease. Therefore, this article examines key gaps in PD care in Cambodia and proposes strategic actions to strengthen the national health system response.

## 2 The Hidden Burden of PD in Cambodia

### 2.1 Absence of Data and Under‐Recognition

Comprehensive surveillance systems for neurological disorders, including PD, are largely absent in many LMICs. In Cambodia, the lack of national registries and population‐based studies limits accurate estimation of disease prevalence, incidence, and outcomes [9]. Consequently, PD remains underrepresented in national health statistics and policy agendas.

The absence of reliable data has significant implications for health system planning and resource allocation [10]. It also contributes to low awareness among healthcare providers and the public, leading to underdiagnosis and misdiagnosis—particularly in rural settings where access to care is limited.

### 2.2 Delayed Diagnosis and Gaps in Primary Care

Primary care serves as the foundation of Cambodia's health system but remains inadequately equipped to detect and manage PD. Similar to other LMICs, patients frequently consult general practitioners with limited training in neurological disorders, increasing the likelihood of delayed or missed diagnoses [11]. Early symptoms—such as tremor, rigidity, and bradykinesia—are often misattributed to normal aging or other conditions.

Delayed diagnosis results in presentation at advanced disease stages, reducing treatment effectiveness and increasing disability. Strengthening primary care through targeted training, simplified diagnostic tools, and effective referral pathways is essential to improve early detection and patient outcomes [5, 12].

### 2.3 Workforce Shortages and Inequitable Access to Care

Cambodia faces a critical shortage of neurologists, as the ratio of Cambodian neurologists to population is 2 per million [13], compared with the American region, which is 7 per million, and Europe, which is 66 per million [14]. Neurologists are concentrated in urban centers such as Phnom Penh; this imbalance results in significant disparities in access to care, particularly for rural populations.

Additionally, PD‐specialized nurses remain the least available and accessible healthcare professionals. Consequently, access to specialized PD care remains disproportionately restricted to wealthier and more influential individuals, further exacerbating health inequities in Cambodia [8]. Specialist neurological services are concentrated primarily in Phnom Penh, requiring many patients from rural and remote areas to travel long distances and incur substantial transportation, accommodation, consultation, diagnostic, and medication costs [15]. Because these expenses are largely financed through out‐of‐pocket (OOP) payments, individuals with higher socioeconomic status are considerably more likely to access specialist care, whereas disadvantaged populations often rely on nonspecialist providers or forgo treatment altogether [16]. This inequitable distribution of neurological services contributes to delayed diagnosis, fragmented care, unnecessary investigations, and suboptimal management of PD.

Evidence from high‐income settings shows that neurologist‐led care is associated with improved outcomes, including reduced hospitalizations and better survival [14, 17]. In Cambodia, however, limited access to specialist care results in fragmented management, inconsistent follow‐up, and poorer health outcomes and reduced quality of life (QoL).

### 2.4 Limited Access to Essential Medicines and Advanced Therapies

Pharmacological treatment, particularly levodopa‐based therapy, remains the cornerstone of PD management [18]. However, access to essential medicines in Cambodia is inconsistent due to supply chain challenges, cost barriers, and limited availability [11]. High OOP expenditures arise from limited insurance coverage for chronic neurological diseases, costs associated with long‐term medications, repeated specialist consultations, transportation to urban referral centers, diagnostic investigations, and rehabilitation services [16]. These financial barriers frequently lead to delayed treatment initiation, poor medication adherence, and interruption of long‐term care.

Although advanced therapies, such as deep brain stimulation (DBS), have become an established treatment option for selected patients with PD worldwide [19], they remain unavailable in Cambodia because of limited infrastructure, specialized expertise, and financial resources [8]. Across Asia, advanced functional neurosurgical procedures, including DBS, are performed primarily in specialized tertiary movement disorder centers in neighboring countries such as Thailand and Vietnam, where well‐established neurology and functional neurosurgery programs exist [20, 21, 22, 23]. Consequently, Cambodian patients who may benefit from DBS must be referred abroad, creating substantial logistical and financial challenges that further limit access to advanced care. These barriers are compounded by the country's severe shortage of movement disorder specialists, the absence of domestic DBS services, and the high OOP costs associated with overseas treatment.

Together, these factors contribute to inequitable access to advanced PD therapies and may result in lower patient satisfaction and poorer long‐term outcomes compared with patients in high‐income countries, where comprehensive multidisciplinary care and advanced treatment options are more readily available.

### 2.5 Neglect of Rehabilitation and Non‐Motor Symptoms

Effective PD management requires a multidisciplinary approach that includes rehabilitation, mental health support, and social services that extend beyond pharmacological treatment [24]. Non‐motor symptoms—including depression, cognitive impairment, and sleep disturbances—are common but frequently under‐recognized in Cambodia, despite their significant impact on QoL [8].

Rehabilitation services, including physiotherapy, speech therapy, and occupational therapy, remain limited and are not routinely integrated into care [25]. Exercise—an essential component of PD management—is often overlooked. The absence of these services contributes to increased disability and caregiver burden. Integrating rehabilitation into primary and community‐based care, alongside strengthening allied health capacity, is essential for comprehensive management.

### 2.6 Barriers to Public Awareness and Health Literacy

Public awareness and health literacy play a critical role in the early recognition and management of PD. However, limited awareness and education of neurological disorders remain a significant challenge in many LMICs, where PD symptoms are frequently misunderstood or attributed to normal aging [25, 26]. In Cambodia, health literacy remains uneven across the population, particularly among older adults and individuals living in rural areas. Low health literacy can hinder individuals’ ability to recognize early symptoms, seek timely medical attention, and adhere to recommended treatments. Consequently, symptoms of PD, such as tremor, rigidity, and slowed movement, are often misunderstood or perceived by patients and their families as normal consequences of aging rather than indicators of a neurological disorder that has a major impact on the QoL among PD patients [26].

Cultural beliefs and stigma surrounding neurological illnesses may further influence care‐seeking behavior [27]. In some communities, symptoms of chronic neurological conditions are attributed to spiritual or supernatural causes [28], which may discourage individuals from seeking formal medical care and lead to delays in diagnosis while increasing reliance on traditional or informal treatment practices.

Additional barriers include the limited availability of PD‐related educational resources in the Khmer language. Patient education materials, support groups, and community outreach initiatives focused on PD remain scarce in Cambodia, and the lack of culturally appropriate health education resources has been widely recognized as a major obstacle to improving disease awareness and patient engagement in many LMICs. Although the International Parkinson and Movement Disorder Society (MDS) provides multilingual patient education resources and professional educational materials, including patient handouts in several languages [29], awareness and utilization of these resources remain limited in Cambodia. Expanding culturally and linguistically appropriate educational materials in the Khmer language is, therefore, essential to improve patient engagement, health literacy, and early care‐seeking behavior. Beyond educational resources, the MDS Center‐to‐Center Movement Disorders Training Program represents an important opportunity to strengthen local clinical capacity in LMICs.

Cambodian hospitals and universities could establish collaborative partnerships with experienced movement disorder centers in neighboring countries, particularly Thailand, Singapore, Malaysia, and Vietnam, through clinician exchanges, mentorship, multidisciplinary training, virtual education, and continuing professional development [30]. Such collaborations would complement locally adapted educational initiatives, strengthen the neurology workforce, and promote sustainable capacity building for PD's care in Cambodia.

### 2.7 Policy Gaps and System Fragmentation

Despite the growing burden of NCDs, PD remains largely absent from Cambodia's national health policies and strategic frameworks. This reflects broader under‐recognition of neurological disorders, with policy attention focused primarily on conditions such as cardiovascular disease, diabetes, and cancer [5, 31]. As a result, PD and other neurological conditions receive limited policy prioritization, which can contribute to fragmented services, insufficient funding, and inadequate planning for long‐term care.

Integrating PD into national NCD strategies is essential to ensure a coordinated and sustainable response. This should include the development of clinical guidelines, incorporation into health financing schemes, and alignment with universal health coverage (UHC) priorities [32]. Recognizing PD as a public health priority would enable more effective resource allocation, improved service planning, and equitable access to care.

## 3 A Call to Action: Strategic Priorities for Cambodia

Addressing the hidden burden of PD in Cambodia will require a coordinated and multi‐level response. Given the country's resource constraints and evolving health system, interventions should prioritize scalability, sustainability, and equity. Strengthening PD care cannot rely on isolated efforts but must be embedded within broader health system reforms, particularly those aligned with UHC and NCD strategies. Collectively, these strategic priorities provide a roadmap for transforming PD care in Cambodia from a fragmented and under‐recognized issue into a coordinated and effective health system response (Table 1).

**TABLE 1:** Strategic priorities for improving Parkinson's disease (PD) care in Cambodia [8, 9, 10, 12, 13, 17, 18, 19, 20, 25, 26, 27, 28, 30, 31, 32, 33, 34].

Priority area	Key actions	Expected impact
Surveillance and data systems	Establish national PD registry; integrate PD into health information systems; promote epidemiological research	Improved understanding of disease burden; evidence‐based planning and policy development
Primary care strengthening	Train primary care providers; develop diagnostic and referral guidelines; implement task‐sharing models	Earlier diagnosis; improved access to care, especially in rural areas
Workforce development	Expand neurology training; build capacity of general practitioners and allied health professionals; foster international partnerships and cross‐cultural education	Increased availability of skilled providers; reduced inequities in access to specialist care
Access to treatment	Include PD drugs in essential medicines list; strengthen procurement and supply chains; establish DBS program; expand financial protection schemes	Improved treatment continuity; reduced OOP costs; better symptom control and diagnosis
Rehabilitation and multidisciplinary care	Integrate physiotherapy, speech therapy, and mental health services; improve referral pathways; develop community‐based rehabilitation programs	Improved functional outcomes; reduced disability and caregiver burden
Technology and digital innovation	Expand telemedicine and teleneurology services; develop electronic PD registries and integrate PD into health information systems; adapt digital health and assistive technologies for local use; promote the responsible use of AI for clinical decision‐making and health planning; develop Khmer‐language digital education resources	Improved access to specialist care; timely diagnosis and referral; strengthened disease surveillance and evidence‐based planning; enhanced patient engagement and continuity of care; more equitable, efficient, and sustainable PD services
Public awareness and health literacy	Conduct national awareness campaigns; develop culturally appropriate educational materials; engage communities	Increased awareness; reduced stigma; earlier care‐seeking behavior
Policy integration and governance	Integrate PD into national NCD strategies and UHC policies; develop national clinical guidelines; establish coordination mechanisms	Stronger policy support; more coordinated and sustainable health system response

Abbreviations: DBS, deep brain stimulation; NCD, noncommunicable disease; OOP, out‐of‐pocket; UHC, universal health coverage.

## 4 Conclusion

PD in Cambodia represents a hidden but growing public health challenge. The absence of data, limited workforce capacity, inequitable access to care, and policy neglect have resulted in substantial unmet needs. Without timely intervention, the burden of PD will continue to rise, disproportionately affecting vulnerable populations. Addressing this challenge requires a shift from invisibility to recognition, from fragmentation to integration and from neglect to action. By prioritizing PD within the national health agenda and implementing targeted, system‐level interventions, Cambodia has the opportunity to improve outcomes for individuals living with PD and move closer toward equitable and comprehensive neurological care.

## Author Contributions

**Virak Sorn**: conceptualization, data curation, formal analysis, writing – original draft, writing – review and editing.

## Funding

The author has nothing to report.

## Ethics Statement

The author has nothing to report.

## Conflicts of Interest

The author declares no conflicts of interest.

## Transparency Statement

The author Virak Sorn affirms that this manuscript is an honest, accurate, and transparent account of the study being reported; that no important aspects of the study have been omitted; and that any discrepancies from the study as planned (and, if relevant, registered) have been explained.

## Data Availability

Due to the fact that no data sets were created or examined for this article, data sharing is not applicable.
